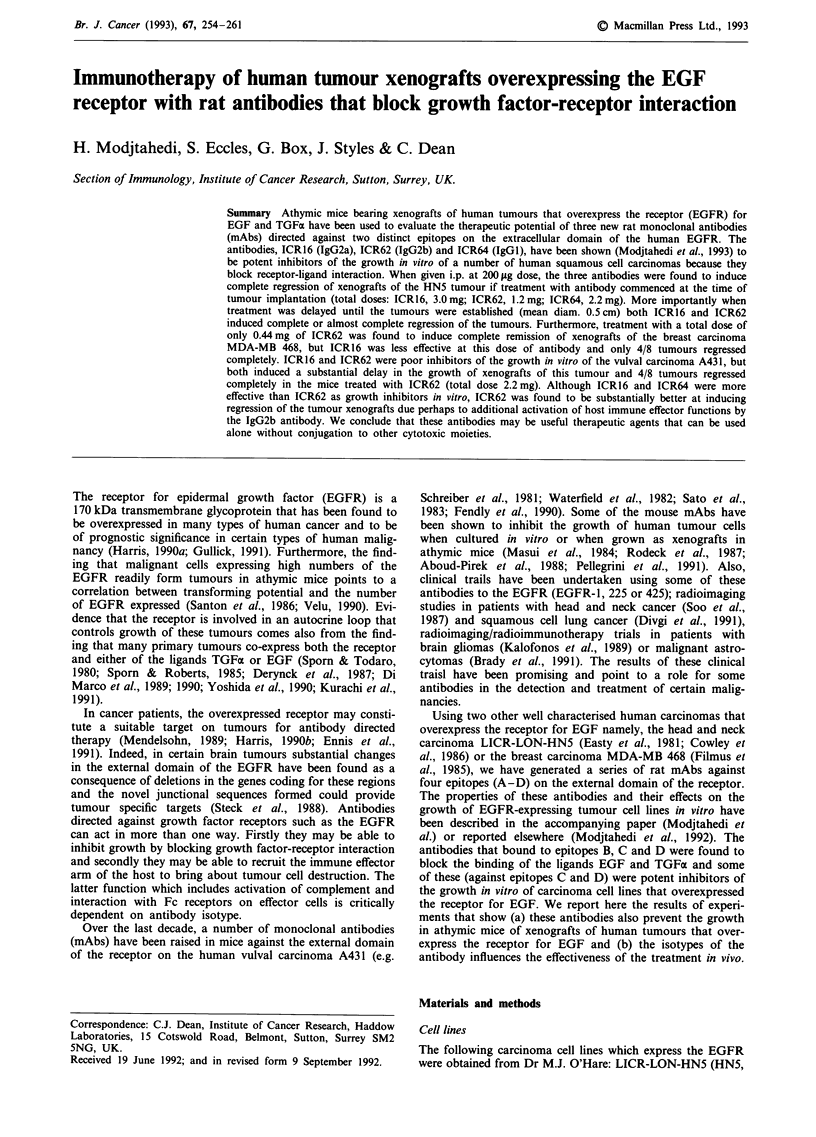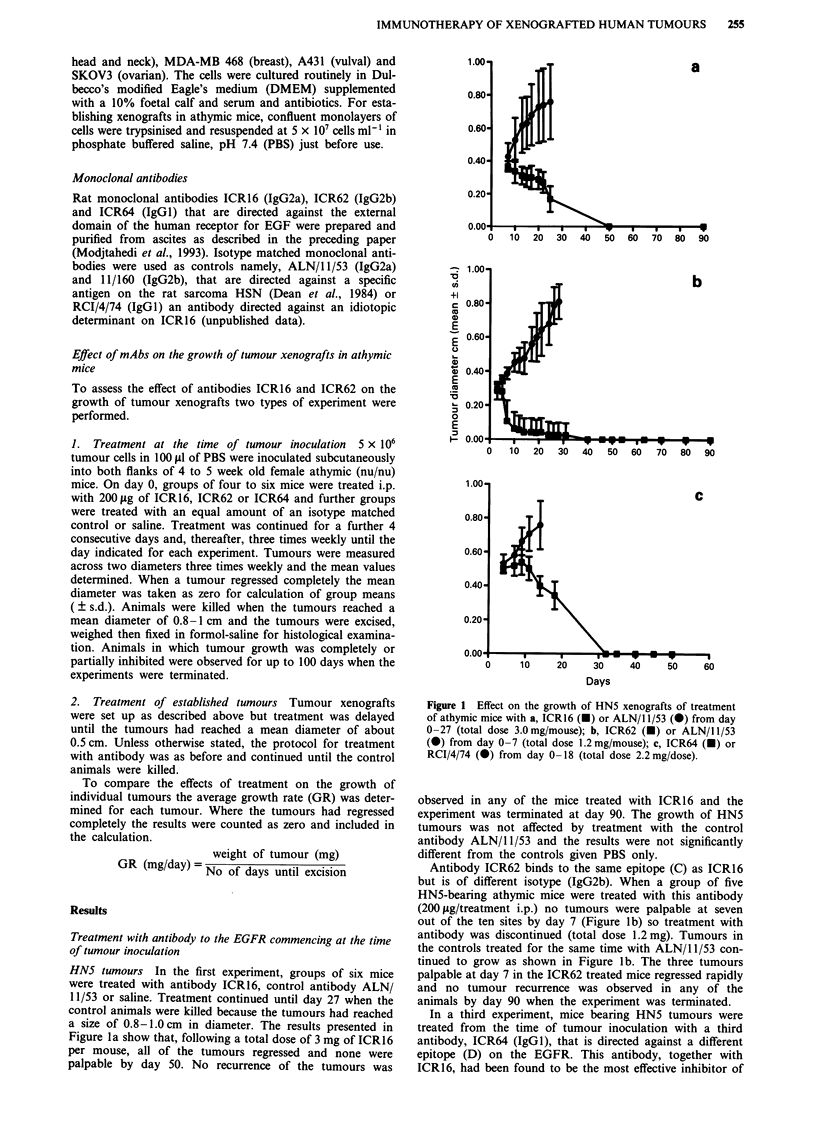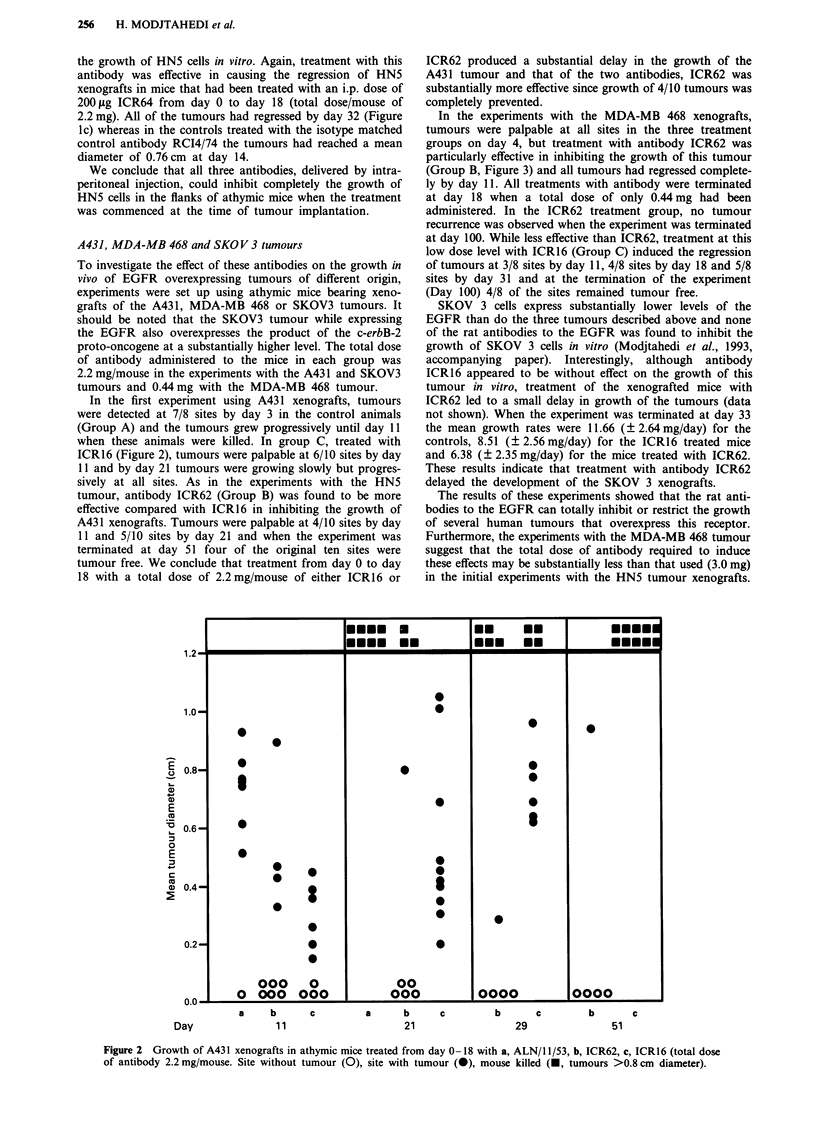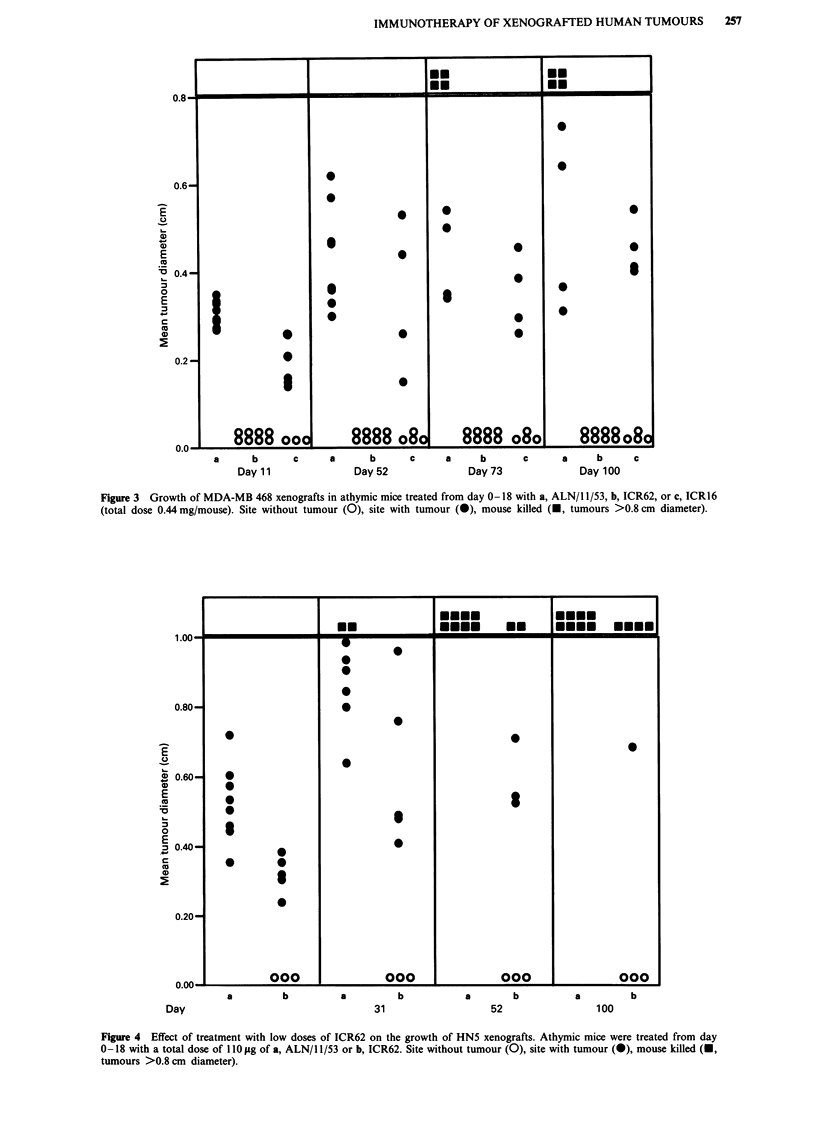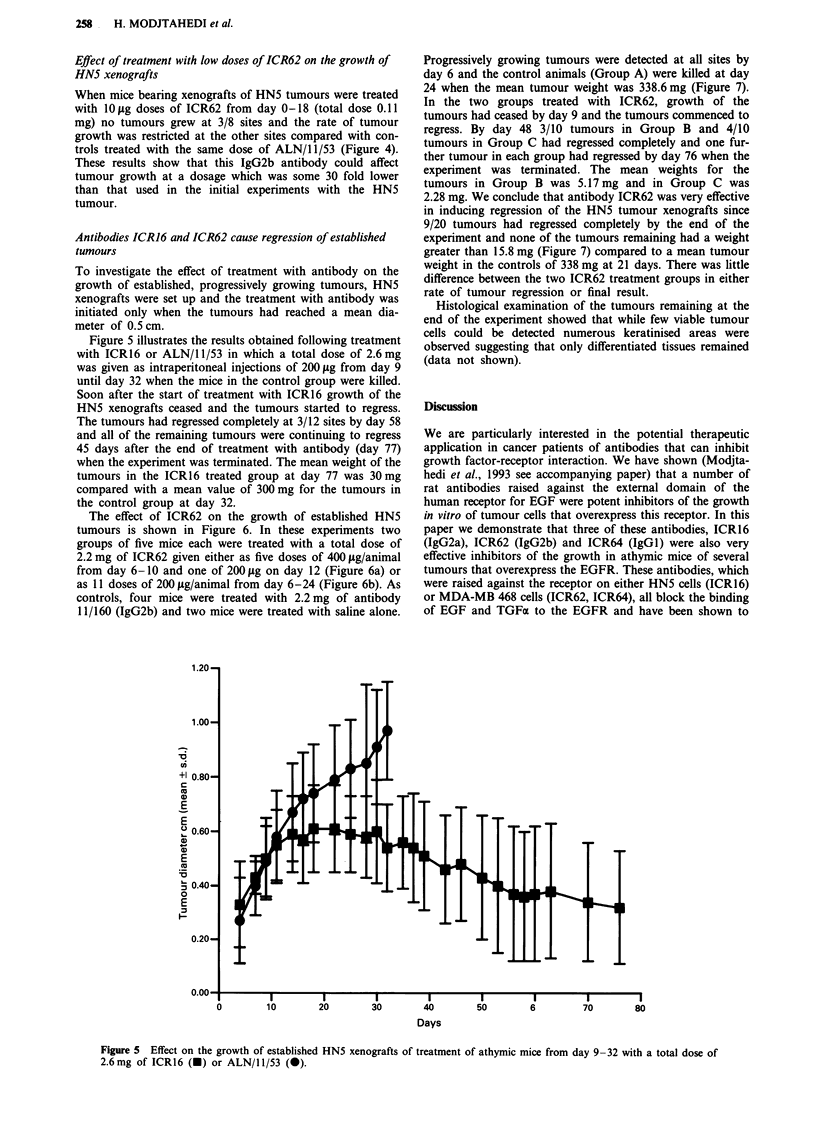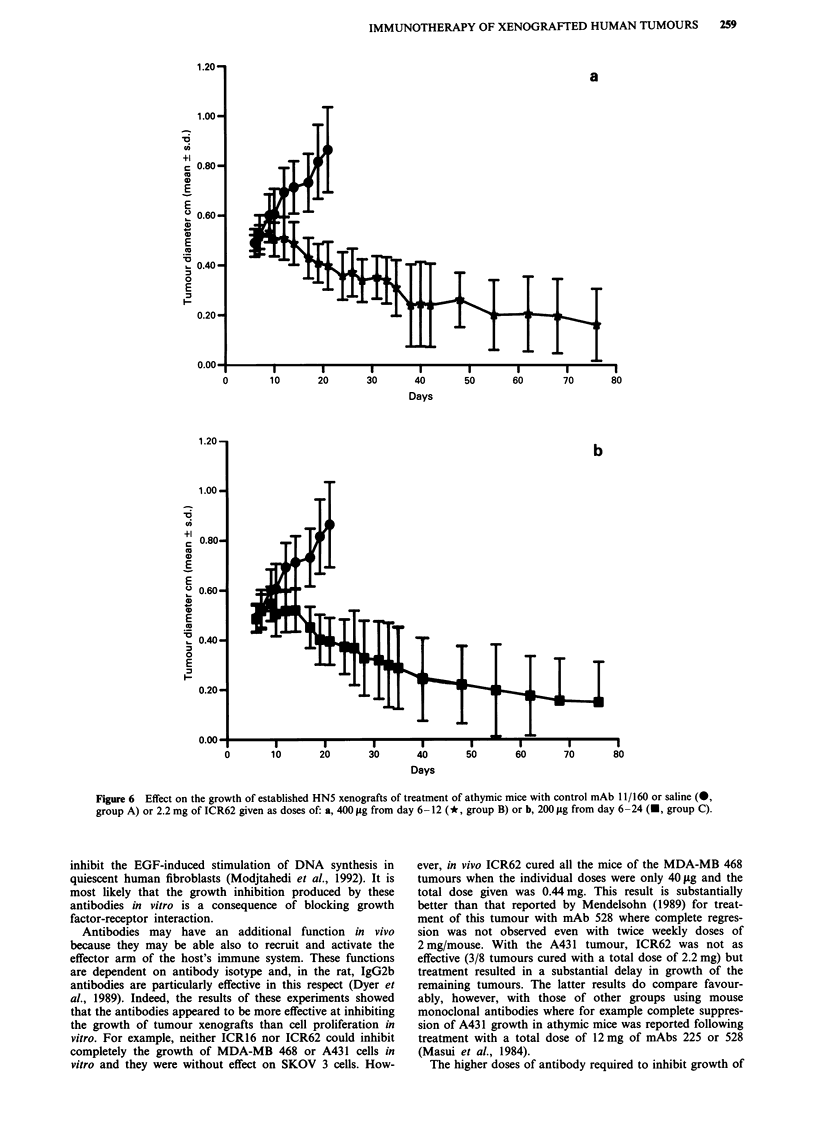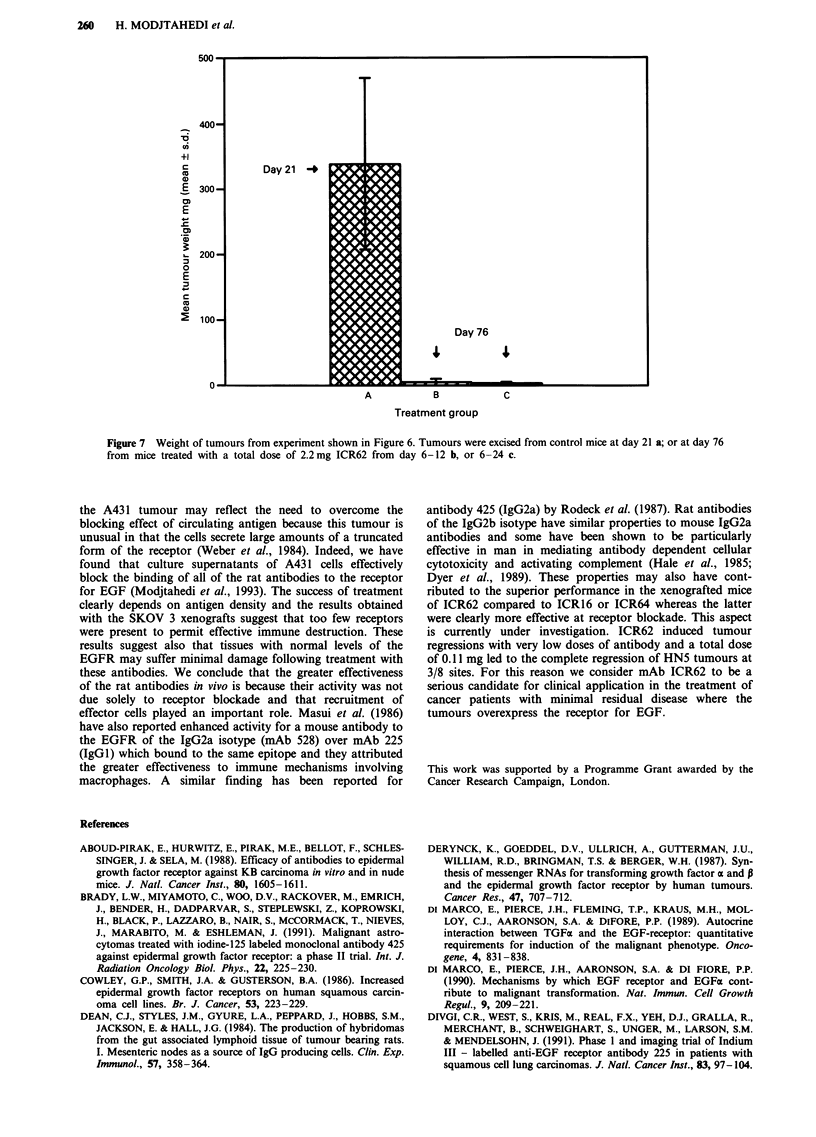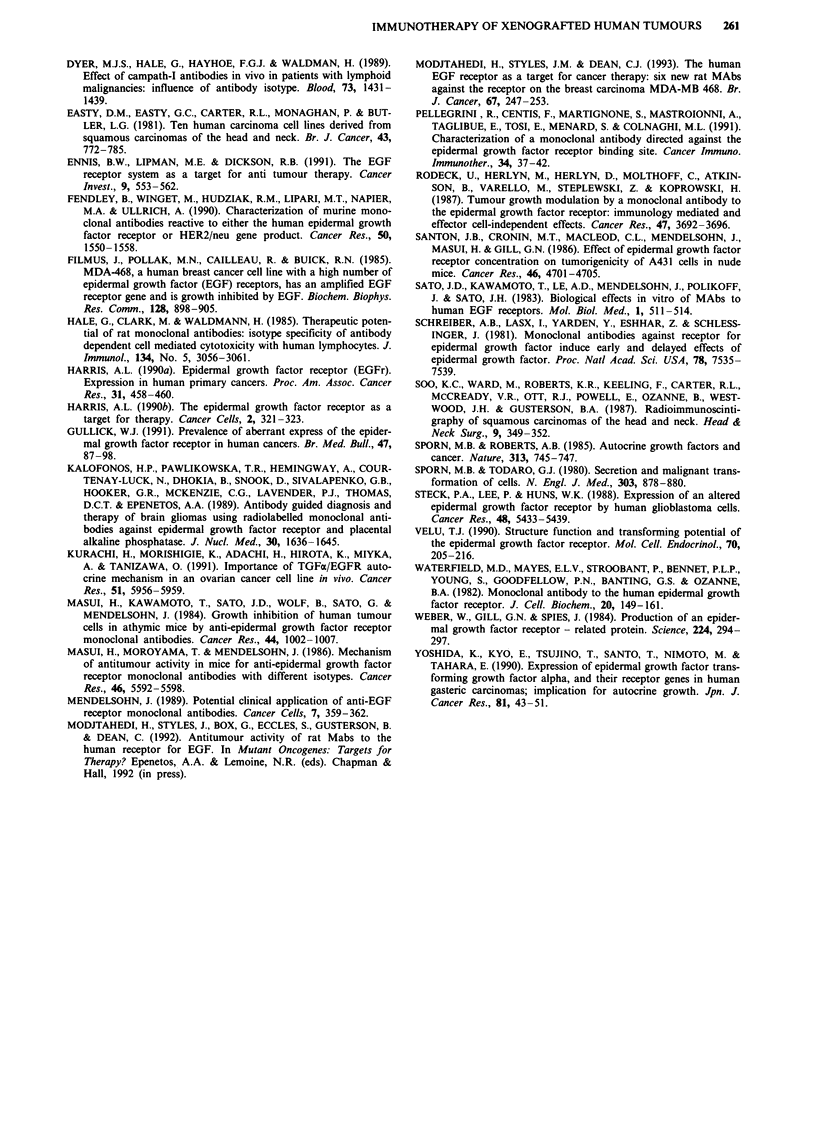# Immunotherapy of human tumour xenografts overexpressing the EGF receptor with rat antibodies that block growth factor-receptor interaction.

**DOI:** 10.1038/bjc.1993.49

**Published:** 1993-02

**Authors:** H. Modjtahedi, S. Eccles, G. Box, J. Styles, C. Dean

**Affiliations:** Section of Immunology, Institute of Cancer Research, Sutton, Surrey, UK.

## Abstract

Athymic mice bearing xenografts of human tumours that overexpress the receptor (EGFR) for EGF and TGF alpha have been used to evaluate the therapeutic potential of three new rat monoclonal antibodies (mAbs) directed against two distinct epitopes on the extracellular domain of the human EGFR. The antibodies, ICR16 (IgG2a), ICR62 (IgG2b) and ICR64 (IgG1), have been shown (Modjtahedi et al., 1993) to be potent inhibitors of the growth in vitro of a number of human squamous cell carcinomas because they block receptor-ligand interaction. When given i.p. at 200 micrograms dose, the three antibodies were found to induce complete regression of xenografts of the HN5 tumour if treatment with antibody commenced at the time of tumour implantation (total doses: ICR16, 3.0 mg; ICR62, 1.2 mg; ICR64, 2.2 mg). More importantly when treatment was delayed until the tumours were established (mean diam. 0.5 cm) both ICR16 and ICR62 induced complete or almost complete regression of the tumours. Furthermore, treatment with a total dose of only 0.44 mg of ICR62 was found to induce complete remission of xenografts of the breast carcinoma MDA-MB 468, but ICR16 was less effective at this dose of antibody and only 4/8 tumours regressed completely. ICR16 and ICR62 were poor inhibitors of the growth in vitro of the vulval carcinoma A431, but both induced a substantial delay in the growth of xenografts of this tumour and 4/8 tumours regressed completely in the mice treated with ICR62 (total dose 2.2 mg). Although ICR16 and ICR64 were more effective than ICR62 as growth inhibitors in vitro, ICR62 was found to be substantially better at inducing regression of the tumour xenografts due perhaps to additional activation of host immune effector functions by the IgG2b antibody. We conclude that these antibodies may be useful therapeutic agents that can be used alone without conjugation to other cytotoxic moieties.


Br. J. Cancer (1993), 67, 254–261 © Macmillan Press Ltd., 1993

### Immunotherapy of human tumour xenografts overexpressing the EGF receptor with rat antibodies that block growth factor-receptor interaction

H. Modjtahedi, S. Eccles, G. Box, J. Styles & C. Dean

*Section of Immunology, Institute of Cancer Research, Sutton, Surrey, UK.*

**Summary** Athymic mice bearing xenografts of human tumours that overexpress the receptor (EGFR) for EGF and TGFα have been used to evaluate the therapeutic potential of three new rat monoclonal antibodies (mAbs) directed against two distinct epitopes on the extracellular domain of the human EGFR. The antibodies, ICR16 (IgG2a), ICR62 (IgG2b) and ICR64 (IgG1), have been shown (Modjtahedi *et al.*, 1993) to be potent inhibitors of the growth *in vitro* of a number of human squamous cell carcinomas because they block receptor-ligand interaction. When given i.p. at 200 μg dose, the three antibodies were found to induce complete regression of xenografts of the HN5 tumour if treatment with antibody commenced at the time of tumour implantation (total doses: ICR16, 3.0 mg; ICR62, 1.2 mg; ICR64, 2.2 mg). More importantly when treatment was delayed until the tumours were established (mean diam. 0.5 cm) both ICR16 and ICR62 induced complete or almost complete regression of the tumours. Furthermore, treatment with a total dose of only 0.44 mg of ICR62 was found to induce complete remission of xenografts of the breast carcinoma MDA-MB 468, but ICR16 was less effective at this dose of antibody and only 4/8 tumours regressed completely. ICR16 and ICR62 were poor inhibitors of the growth *in vitro* of the vulval carcinoma A431, but both induced a substantial delay in the growth of xenografts of this tumour and 4/8 tumours regressed completely in the mice treated with ICR62 (total dose 2.2 mg). Although ICR16 and ICR64 were more effective than ICR62 as growth inhibitors *in vitro*, ICR62 was found to be substantially better at inducing regression of the tumour xenografts due perhaps to additional activation of host immune effector functions by the IgG2b antibody. We conclude that these antibodies may be useful therapeutic agents that can be used alone without conjugation to other cytotoxic moieties.

The receptor for epidermal growth factor (EGFR) is a 170 kDa transmembrane glycoprotein that has been found to be overexpressed in many types of human cancer and to be of prognostic significance in certain types of human malignancy (Harris, 1990*a*; Gullick, 1991). Furthermore, the finding that malignant cells expressing high numbers of the EGFR readily form tumours in athymic mice points to a correlation between transforming potential and the number of EGFR expressed (Santon *et al.*, 1986; Velu, 1990). Evidence that the receptor is involved in an autocrine loop that controls growth of these tumours comes also from the finding that many primary tumours co-express both the receptor and either of the ligands TGFα or EGF (Sporn & Todaro, 1980; Sporn & Roberts, 1985; Derynck *et al.*, 1987; Di Marco *et al.*, 1989; 1990; Yoshida *et al.*, 1990; Kurachi *et al.*, 1991).

In cancer patients, the overexpressed receptor may constitute a suitable target on tumours for antibody directed therapy (Mendelsohn, 1989; Harris, 1990*b*; Ennis *et al.*, 1991). Indeed, in certain brain tumours substantial changes in the external domain of the EGFR have been found as a consequence of deletions in the genes coding for these regions and the novel junctional sequences formed could provide tumour specific targets (Steck *et al.*, 1988). Antibodies directed against growth factor receptors such as the EGFR can act in more than one way. Firstly they may be able to inhibit growth by blocking growth factor-receptor interaction and secondly they may be able to recruit the immune effector arm of the host to bring about tumour cell destruction. The latter function which includes activation of complement and interaction with Fc receptors on effector cells is critically dependent on antibody isotype.

Over the last decade, a number of monoclonal antibodies (mAbs) have been raised in mice against the external domain of the receptor on the human vulval carcinoma A431 (e.g. Schreiber *et al.*, 1981; Waterfield *et al.*, 1982; Sato *et al.*, 1983; Fendly *et al.*, 1990). Some of the mouse mAbs have been shown to inhibit the growth of human tumour cells when cultured *in vitro* or when grown as xenografts in athymic mice (Masui *et al.*, 1984; Rodeck *et al.*, 1987; Aboud-Pirek *et al.*, 1988; Pellegrini *et al.*, 1991). Also, clinical trails have been undertaken using some of these antibodies to the EGFR (EGFR-1, 225 or 425); radioimaging studies in patients with head and neck cancer (Soo *et al.*, 1987) and squamous cell lung cancer (Divgi *et al.*, 1991), radioimaging/radioimmunotherapy trials in patients with brain gliomas (Kalofonos *et al.*, 1989) or malignant astrocytomas (Brady *et al.*, 1991). The results of these clinical traisl have been promising and point to a role for some antibodies in the detection and treatment of certain malignancies.

Using two other well characterised human carcinomas that overexpress the receptor for EGF namely, the head and neck carcinoma LICR-LON-HN5 (Easty *et al.*, 1981; Cowley *et al.*, 1986) or the breast carcinoma MDA-MB 468 (Filmus *et al.*, 1985), we have generated a series of rat mAbs against four epitopes (A–D) on the external domain of the receptor. The properties of these antibodies and their effects on the growth of EGFR-expressing tumour cell lines *in vitro* have been described in the accompanying paper (Modjtahedi *et al.*) or reported elsewhere (Modjtahedi *et al.*, 1992). The antibodies that bound to epitopes B, C and D were found to block the binding of the ligands EGF and TGFα and some of these (against epitopes C and D) were potent inhibitors of the growth *in vitro* of carcinoma cell lines that overexpressed the receptor for EGF. We report here the results of experiments that show (a) these antibodies also prevent the growth in athymic mice of xenografts of human tumours that overexpress the receptor for EGF and (b) the isotypes of the antibody influences the effectiveness of the treatment *in vivo*.

### Materials and methods

### *Cell lines*

The following carcinoma cell lines which express the EGFR were obtained from Dr M.J. O'Hare: LICR-LON-HN5 (HN5,

Correspondence: C.J. Dean, Institute of Cancer Research, Haddow Laboratories, 15 Cotswold Road, Belmont, Sutton, Surrey SM2 5NG, UK.
Received 19 June 1992; and in revised form 9 September 1992.

head and neck), MDA-MB 468 (breast), A431 (vulval) and SKOV3 (ovarian). The cells were cultured routinely in Dulbecco's modified Eagle's medium (DMEM) supplemented with a 10% foetal calf and serum and antibiotics. For establishing xenografts in athymic mice, confluent monolayers of cells were trypsinised and resuspended at $5 \times 10^7$ cells $ml^{-1}$ in phosphate buffered saline, pH 7.4 (PBS) just before use.

*Monoclonal antibodies*

Rat monoclonal antibodies ICR16 (IgG2a), ICR62 (IgG2b) and ICR64 (IgG1) that are directed against the external domain of the human receptor for EGF were prepared and purified from ascites as described in the preceding paper (Modjtahedi *et al.*, 1993). Isotype matched monoclonal antibodies were used as controls namely, ALN/11/53 (IgG2a) and 11/160 (IgG2b), that are directed against a specific antigen on the rat sarcoma HSN (Dean *et al.*, 1984) or RCI/4/74 (IgG1) an antibody directed against an idiotopic determinant on ICR16 (unpublished data).

*Effect of mAbs on the growth of tumour xenografts in athymic mice*

To assess the effect of antibodies ICR16 and ICR62 on the growth of tumour xenografts two types of experiment were performed.

1. *Treatment at the time of tumour inoculation* $5 \times 10^6$ tumour cells in 100 µl of PBS were inoculated subcutaneously into both flanks of 4 to 5 week old female athymic (nu/nu) mice. On day 0, groups of four to six mice were treated i.p. with 200 µg of ICR16, ICR62 or ICR64 and further groups were treated with an equal amount of an isotype matched control or saline. Treatment was continued for a further 4 consecutive days and, thereafter, three times weekly until the day indicated for each experiment. Tumours were measured across two diameters three times weekly and the mean values determined. When a tumour regressed completely the mean diameter was taken as zero for calculation of group means (± s.d.). Animals were killed when the tumours reached a mean diameter of 0.8–1 cm and the tumours were excised, weighed then fixed in formol-saline for histological examination. Animals in which tumour growth was completely or partially inhibited were observed for up to 100 days when the experiments were terminated.

2. *Treatment of established tumours* Tumour xenografts were set up as described above but treatment was delayed until the tumours had reached a mean diameter of about 0.5 cm. Unless otherwise stated, the protocol for treatment with antibody was as before and continued until the control animals were killed.

To compare the effects of treatment on the growth of individual tumours the average growth rate (GR) was determined for each tumour. Where the tumours had regressed completely the results were counted as zero and included in the calculation.

$$\text{GR (mg/day)} = \frac{\text{weight of tumour (mg)}}{\text{No of days until excision}}$$

### Results

*Treatment with antibody to the EGFR commencing at the time of tumour inoculation*

*HN5 tumours* In the first experiment, groups of six mice were treated with antibody ICR16, control antibody ALN/11/53 or saline. Treatment continued until day 27 when the control animals were killed because the tumours had reached a size of 0.8–1.0 cm in diameter. The results presented in Figure 1a show that, following a total dose of 3 mg of ICR16 per mouse, all of the tumours regressed and none were palpable by day 50. No recurrence of the tumours was

**Figure 1** Effect on the growth of HN5 xenografts of treatment of athymic mice with **a**, ICR16 (■) or ALN/11/53 (●) from day 0–27 (total dose 3.0 mg/mouse); **b**, ICR62 (■) or ALN/11/53 (●) from day 0–7 (total dose 1.2 mg/mouse); **c**, ICR64 (■) or RCI/4/74 (●) from day 0–18 (total dose 2.2 mg/dose).

observed in any of the mice treated with ICR16 and the experiment was terminated at day 90. The growth of HN5 tumours was not affected by treatment with the control antibody ALN/11/53 and the results were not significantly different from the controls given PBS only.

Antibody ICR62 binds to the same epitope (C) as ICR16 but is of different isotype (IgG2b). When a group of five HN5-bearing athymic mice were treated with this antibody (200 µg/treatment i.p.) no tumours were palpable at seven out of the ten sites by day 7 (Figure 1b) so treatment with antibody was discontinued (total dose 1.2 mg). Tumours in the controls treated for the same time with ALN/11/53 continued to grow as shown in Figure 1b. The three tumours palpable at day 7 in the ICR62 treated mice regressed rapidly and no tumour recurrence was observed in any of the animals by day 90 when the experiment was terminated.

In a third experiment, mice bearing HN5 tumours were treated from the time of tumour inoculation with a third antibody, ICR64 (IgG1), that is directed against a different epitope (D) on the EGFR. This antibody, together with ICR16, had been found to be the most effective inhibitor of the growth of HN5 cells *in vitro*. Again, treatment with this antibody was effective in causing the regression of HN5 xenografts in mice that had been treated with an i.p. dose of 200 μg ICR64 from day 0 to day 18 (total dose/mouse of 2.2 mg). All of the tumours had regressed by day 32 (Figure 1c) whereas in the controls treated with the isotype matched control antibody RCI4/74 the tumours had reached a mean diameter of 0.76 cm at day 14.

We conclude that all three antibodies, delivered by intraperitoneal injection, could inhibit completely the growth of HN5 cells in the flanks of athymic mice when the treatment was commenced at the time of tumour implantation.

### *A431, MDA-MB 468 and SKOV 3 tumours*

To investigate the effect of these antibodies on the growth *in vivo* of EGFR overexpressing tumours of different origin, experiments were set up using athymic mice bearing xenografts of the A431, MDA-MB 468 or SKOV3 tumours. It should be noted that the SKOV3 tumour while expressing the EGFR also overexpresses the product of the c-*erb*B-2 proto-oncogene at a substantially higher level. The total dose of antibody administered to the mice in each group was 2.2 mg/mouse in the experiments with the A431 and SKOV3 tumours and 0.44 mg with the MDA-MB 468 tumour.

In the first experiment using A431 xenografts, tumours were detected at 7/8 sites by day 3 in the control animals (Group A) and the tumours grew progressively until day 11 when these animals were killed. In group C, treated with ICR16 (Figure 2), tumours were palpable at 6/10 sites by day 11 and by day 21 tumours were growing slowly but progressively at all sites. As in the experiments with the HN5 tumour, antibody ICR62 (Group B) was found to be more effective compared with ICR16 in inhibiting the growth of A431 xenografts. Tumours were palpable at 4/10 sites by day 11 and 5/10 sites by day 21 and when the experiment was terminated at day 51 four of the original ten sites were tumour free. We conclude that treatment from day 0 to day 18 with a total dose of 2.2 mg/mouse of either ICR16 or ICR62 produced a substantial delay in the growth of the A431 tumour and that of the two antibodies, ICR62 was substantially more effective since growth of 4/10 tumours was completely prevented.

In the experiments with the MDA-MB 468 xenografts, tumours were palpable at all sites in the three treatment groups on day 4, but treatment with antibody ICR62 was particularly effective in inhibiting the growth of this tumour (Group B, Figure 3) and all tumours had regressed completely by day 11. All treatments with antibody were terminated at day 18 when a total dose of only 0.44 mg had been administered. In the ICR62 treatment group, no tumour recurrence was observed when the experiment was terminated at day 100. While less effective than ICR62, treatment at this low dose level with ICR16 (Group C) induced the regression of tumours at 3/8 sites by day 11, 4/8 sites by day 18 and 5/8 sites by day 31 and at the termination of the experiment (Day 100) 4/8 of the sites remained tumour free.

SKOV 3 cells express substantially lower levels of the EGFR than do the three tumours described above and none of the rat antibodies to the EGFR was found to inhibit the growth of SKOV 3 cells *in vitro* (Modjtahedi *et al.*, 1993, accompanying paper). Interestingly, although antibody ICR16 appeared to be without effect on the growth of this tumour *in vitro*, treatment of the xenografted mice with ICR62 led to a small delay in growth of the tumours (data not shown). When the experiment was terminated at day 33 the mean growth rates were 11.66 (± 2.64 mg/day) for the controls, 8.51 (± 2.56 mg/day) for the ICR16 treated mice and 6.38 (± 2.35 mg/day) for the mice treated with ICR62. These results indicate that treatment with antibody ICR62 delayed the development of the SKOV 3 xenografts.

The results of these experiments showed that the rat antibodies to the EGFR can totally inhibit or restrict the growth of several human tumours that overexpress this receptor. Furthermore, the experiments with the MDA-MB 468 tumour suggest that the total dose of antibody required to induce these effects may be substantially less than that used (3.0 mg) in the initial experiments with the HN5 tumour xenografts.

**Figure 2** Growth of A431 xenografts in athymic mice treated from day 0–18 with **a**, ALN/11/53, **b**, ICR62, **c**, ICR16 (total dose of antibody 2.2 mg/mouse. Site without tumour (O), site with tumour (●), mouse killed (■, tumours >0.8 cm diameter).

**Figure 3** Growth of MDA-MB 468 xenografts in athymic mice treated from day 0–18 with **a**, ALN/11/53, **b**, ICR62, or **c**, ICR16 (total dose 0.44 mg/mouse). Site without tumour (○), site with tumour (●), mouse killed (■, tumours >0.8 cm diameter).

**Figure 4** Effect of treatment with low doses of ICR62 on the growth of HN5 xenografts. Athymic mice were treated from day 0–18 with a total dose of 110 μg of **a**, ALN/11/53 or **b**, ICR62. Site without tumour (○), site with tumour (●), mouse killed (■, tumours >0.8 cm diameter).

*Effect of treatment with low doses of ICR62 on the growth of HN5 xenografts*

When mice bearing xenografts of HN5 tumours were treated with 10 μg doses of ICR62 from day 0–18 (total dose 0.11 mg) no tumours grew at 3/8 sites and the rate of tumour growth was restricted at the other sites compared with controls treated with the same dose of ALN/11/53 (Figure 4). These results show that this IgG2b antibody could affect tumour growth at a dosage which was some 30 fold lower than that used in the initial experiments with the HN5 tumour.

*Antibodies ICR16 and ICR62 cause regression of established tumours*

To investigate the effect of treatment with antibody on the growth of established, progressively growing tumours, HN5 xenografts were set up and the treatment with antibody was initiated only when the tumours had reached a mean diameter of 0.5 cm.

Figure 5 illustrates the results obtained following treatment with ICR16 or ALN/11/53 in which a total dose of 2.6 mg was given as intraperitoneal injections of 200 μg from day 9 until day 32 when the mice in the control group were killed. Soon after the start of treatment with ICR16 growth of the HN5 xenografts ceased and the tumours started to regress. The tumours had regressed completely at 3/12 sites by day 58 and all of the remaining tumours were continuing to regress 45 days after the end of treatment with antibody (day 77) when the experiment was terminated. The mean weight of the tumours in the ICR16 treated group at day 77 was 30 mg compared with a mean value of 300 mg for the tumours in the control group at day 32.

The effect of ICR62 on the growth of established HN5 tumours is shown in Figure 6. In these experiments two groups of five mice each were treated with a total dose of 2.2 mg of ICR62 given either as five doses of 400 μg/animal from day 6–10 and one of 200 μg on day 12 (Figure 6a) or as 11 doses of 200 μg/animal from day 6–24 (Figure 6b). As controls, four mice were treated with 2.2 mg of antibody 11/160 (IgG2b) and two mice were treated with saline alone. Progressively growing tumours were detected at all sites by day 6 and the control animals (Group A) were killed at day 24 when the mean tumour weight was 338.6 mg (Figure 7). In the two groups treated with ICR62, growth of the tumours had ceased by day 9 and the tumours commenced to regress. By day 48 3/10 tumours in Group B and 4/10 tumours in Group C had regressed completely and one further tumour in each group had regressed by day 76 when the experiment was terminated. The mean weights for the tumours in Group B was 5.17 mg and in Group C was 2.28 mg. We conclude that antibody ICR62 was very effective in inducing regression of the HN5 tumour xenografts since 9/20 tumours had regressed completely by the end of the experiment and none of the tumours remaining had a weight greater than 15.8 mg (Figure 7) compared to a mean tumour weight in the controls of 338 mg at 21 days. There was little difference between the two ICR62 treatment groups in either rate of tumour regression or final result.

Histological examination of the tumours remaining at the end of the experiment showed that while few viable tumour cells could be detected numerous keratinised areas were observed suggesting that only differentiated tissues remained (data not shown).

### Discussion

We are particularly interested in the potential therapeutic application in cancer patients of antibodies that can inhibit growth factor-receptor interaction. We have shown (Modjtahedi *et al.*, 1993 see accompanying paper) that a number of rat antibodies raised against the external domain of the human receptor for EGF were potent inhibitors of the growth *in vitro* of tumour cells that overexpress this receptor. In this paper we demonstrate that three of these antibodies, ICR16 (IgG2a), ICR62 (IgG2b) and ICR64 (IgG1) were also very effective inhibitors of the growth in athymic mice of several tumours that overexpress the EGFR. These antibodies, which were raised against the receptor on either HN5 cells (ICR16) or MDA-MB 468 cells (ICR62, ICR64), all block the binding of EGF and TGFα to the EGFR and have been shown to

**Figure 5** Effect on the growth of established HN5 xenografts of treatment of athymic mice from day 9–32 with a total dose of 2.6 mg of ICR16 (■) or ALN/11/53 (●).

**Figure 6** Effect on the growth of established HN5 xenografts of treatment of athymic mice with control mAb 11/160 or saline (●, group A) or 2.2 mg of ICR62 given as doses of: **a**, 400 μg from day 6–12 (★, group B) or **b**, 200 μg from day 6–24 (■, group C).

inhibit the EGF-induced stimulation of DNA synthesis in quiescent human fibroblasts (Modjtahedi *et al.*, 1992). It is most likely that the growth inhibition produced by these antibodies *in vitro* is a consequence of blocking growth factor-receptor interaction.

Antibodies may have an additional function *in vivo* because they may be able also to recruit and activate the effector arm of the host's immune system. These functions are dependent on antibody isotype and, in the rat, IgG2b antibodies are particularly effective in this respect (Dyer *et al.*, 1989). Indeed, the results of these experiments showed that the antibodies appeared to be more effective at inhibiting the growth of tumour xenografts than cell proliferation *in vitro*. For example, neither ICR16 nor ICR62 could inhibit completely the growth of MDA-MB 468 or A431 cells *in vitro* and they were without effect on SKOV 3 cells. However, *in vivo* ICR62 cured all the mice of the MDA-MB 468 tumours when the individual doses were only 40 μg and the total dose given was 0.44 mg. This result is substantially better than that reported by Mendelsohn (1989) for treatment of this tumour with mAb 528 where complete regression was not observed even with twice weekly doses of 2 mg/mouse. With the A431 tumour, ICR62 was not as effective (3/8 tumours cured with a total dose of 2.2 mg) but treatment resulted in a substantial delay in growth of the remaining tumours. The latter results do compare favourably, however, with those of other groups using mouse monoclonal antibodies where for example complete suppression of A431 growth in athymic mice was reported following treatment with a total dose of 12 mg of mAbs 225 or 528 (Masui *et al.*, 1984).

The higher doses of antibody required to inhibit growth of

**Figure 7** Weight of tumours from experiment shown in Figure 6. Tumours were excised from control mice at day 21 **a**; or at day 76 from mice treated with a total dose of 2.2 mg ICR62 from day 6–12 **b**, or 6–24 **c**.

the A431 tumour may reflect the need to overcome the blocking effect of circulating antigen because this tumour is unusual in that the cells secrete large amounts of a truncated form of the receptor (Weber *et al.*, 1984). Indeed, we have found that culture supernatants of A431 cells effectively block the binding of all of the rat antibodies to the receptor for EGF (Modjtahedi *et al.*, 1993). The success of treatment clearly depends on antigen density and the results obtained with the SKOV 3 xenografts suggest that too few receptors were present to permit effective immune destruction. These results suggest also that tissues with normal levels of the EGFR may suffer minimal damage following treatment with these antibodies. We conclude that the greater effectiveness of the rat antibodies *in vivo* is because their activity was not due solely to receptor blockade and that recruitment of effector cells played an important role. Masui *et al.* (1986) have also reported enhanced activity for a mouse antibody to the EGFR of the IgG2a isotype (mAb 528) over mAb 225 (IgG1) which bound to the same epitope and they attributed the greater effectiveness to immune mechanisms involving macrophages. A similar finding has been reported for antibody 425 (IgG2a) by Rodeck *et al.* (1987). Rat antibodies of the IgG2b isotype have similar properties to mouse IgG2a antibodies and some have been shown to be particularly effective in man in mediating antibody dependent cellular cytotoxicity and activating complement (Hale *et al.*, 1985; Dyer *et al.*, 1989). These properties may also have contributed to the superior performance in the xenografted mice of ICR62 compared to ICR16 or ICR64 whereas the latter were clearly more effective at receptor blockade. This aspect is currently under investigation. ICR62 induced tumour regressions with very low doses of antibody and a total dose of 0.11 mg led to the complete regression of HN5 tumours at 3/8 sites. For this reason we consider mAb ICR62 to be a serious candidate for clinical application in the treatment of cancer patients with minimal residual disease where the tumours overexpress the receptor for EGF.

This work was supported by a Programme Grant awarded by the Cancer Research Campaign, London.

### References

ABOUD-PIRAK, E., HURWITZ, E., PIRAK, M.E., BELLOT, F., SCHLESSINGER, J. & SELA, M. (1988). Efficacy of antibodies to epidermal growth factor receptor against KB carcinoma *in vitro* and in nude mice. *J. Natl. Cancer Inst.*, **80**, 1605–1611.

BRADY, L.W., MIYAMOTO, C., WOO, D.V., RACKOVER, M., EMRICH, J., BENDER, H., DADPARVAR, S., STEPLEWSKI, Z., KOPROWSKI, H., BLACK, P., LAZZARO, B., NAIR, S., MCCORMACK, T., NIEVES, J., MARABITO, M. & ESHLEMAN, J. (1991). Malignant astrocytomas treated with iodine-125 labeled monoclonal antibody 425 against epidermal growth factor receptor: a phase II trial. *Int. J. Radiation Oncology Biol. Phys.*, **22**, 225–230.

COWLEY, G.P., SMITH, J.A. & GUSTERSON, B.A. (1986). Increased epidermal growth factor receptors on human squamous carcinoma cell lines. *Br. J. Cancer*, **53**, 223–229.

DEAN, C.J., STYLES, J.M., GYURE, L.A., PEPPARD, J., HOBBS, S.M., JACKSON, E. & HALL, J.G. (1984). The production of hybridomas from the gut associated lymphoid tissue of tumour bearing rats. I. Mesenteric nodes as a source of IgG producing cells. *Clin. Exp. Immunol.*, **57**, 358–364.

DERYNCK, K., GOEDDEL, D.V., ULLRICH, A., GUTTERMAN, J.U., WILLIAM, R.D., BRINGMAN, T.S. & BERGER, W.H. (1987). Synthesis of messenger RNAs for transforming growth factor α and β and the epidermal growth factor receptor by human tumours. *Cancer Res.*, **47**, 707–712.

DI MARCO, E., PIERCE, J.H., FLEMING, T.P., KRAUS, M.H., MOLLOY, C.J., AARONSON, S.A. & DIFORE, P.P. (1989). Autocrine interaction between TGFα and the EGF-receptor: quantitative requirements for induction of the malignant phenotype. *Oncogene*, **4**, 831–838.

DI MARCO, E., PIERCE, J.H., AARONSON, S.A. & DI FIORE, P.P. (1990). Mechanisms by which EGF receptor and EGFα contribute to malignant transformation. *Nat. Immun. Cell Growth Regul.*, **9**, 209–221.

DIVGI, C.R., WEST, S., KRIS, M., REAL, F.X., YEH, D.J., GRALLA, R., MERCHANT, B., SCHWEIGHART, S., UNGER, M., LARSON, S.M. & MENDELSOHN, J. (1991). Phase 1 and imaging trial of Indium III – labelled anti-EGF receptor antibody 225 in patients with squamous cell lung carcinomas. *J. Natl. Cancer Inst.*, **83**, 97–104.

DYER, M.J.S., HALE, G., HAYHOE, F.G.J. & WALDMAN, H. (1989). Effect of campath-I antibodies in vivo in patients with lymphoid malignancies: influence of antibody isotype. *Blood*, **73**, 1431–1439.

EASTY, D.M., EASTY, G.C., CARTER, R.L., MONAGHAN, P. & BUTLER, L.G. (1981). Ten human carcinoma cell lines derived from squamous carcinomas of the head and neck. *Br. J. Cancer*, **43**, 772–785.

ENNIS, B.W., LIPMAN, M.E. & DICKSON, R.B. (1991). The EGF receptor system as a target for anti tumour therapy. *Cancer Invest.*, **9**, 553–562.

FENDLEY, B., WINGET, M., HUDZIAK, R.M., LIPARI, M.T., NAPIER, M.A. & ULLRICH, A. (1990). Characterization of murine monoclonal antibodies reactive to either the human epidermal growth factor receptor or HER2/neu gene product. *Cancer Res.*, **50**, 1550–1558.

FILMUS, J., POLLAK, M.N., CAILLEAU, R. & BUICK, R.N. (1985). MDA-468, a human breast cancer cell line with a high number of epidermal growth factor (EGF) receptors, has an amplified EGF receptor gene and is growth inhibited by EGF. *Biochem. Biophys. Res. Comm.*, **128**, 898–905.

HALE, G., CLARK, M. & WALDMANN, H. (1985). Therapeutic potential of rat monoclonal antibodies: isotype specificity of antibody dependent cell mediated cytotoxicity with human lymphocytes. *J. Immunol.*, **134**, No. 5, 3056–3061.

HARRIS, A.L. (1990*a*). Epidermal growth factor receptor (EGFr). Expression in human primary cancers. *Proc. Am. Assoc. Cancer Res.*, **31**, 458–460.

HARRIS, A.L. (1990*b*). The epidermal growth factor receptor as a target for therapy. *Cancer Cells*, **2**, 321–323.

GULLICK, W.J. (1991). Prevalence of aberrant express of the epidermal growth factor receptor in human cancers. *Br. Med. Bull.*, **47**, 87–98.

KALOFONOS, H.P., PAWLIKOWSKA, T.R., HEMINGWAY, A., COURTENAY-LUCK, N., DHOKIA, B., SNOOK, D., SIVALAPENKO, G.B., HOOKER, G.R., MCKENZIE, C.G., LAVENDER, P.J., THOMAS, D.C.T. & EPENETOS, A.A. (1989). Antibody guided diagnosis and therapy of brain gliomas using radiolabelled monoclonal antibodies against epidermal growth factor receptor and placental alkaline phosphatase. *J. Nucl. Med.*, **30**, 1636–1645.

KURACHI, H., MORISHIGIE, K., ADACHI, H., HIROTA, K., MIYKA, A. & TANIZAWA, O. (1991). Importance of TGFα/EGFR autocrine mechanism in an ovarian cancer cell line *in vivo*. *Cancer Res.*, **51**, 5956–5959.

MASUI, H., KAWAMOTO, T., SATO, J.D., WOLF, B., SATO, G. & MENDELSOHN, J. (1984). Growth inhibition of human tumour cells in athymic mice by anti-epidermal growth factor receptor monoclonal antibodies. *Cancer Res.*, **44**, 1002–1007.

MASUI, H., MOROYAMA, T. & MENDELSOHN, J. (1986). Mechanism of antitumour activity in mice for anti-epidermal growth factor receptor monoclonal antibodies with different isotypes. *Cancer Res.*, **46**, 5592–5598.

MENDELSOHN, J. (1989). Potential clinical application of anti-EGF receptor monoclonal antibodies. *Cancer Cells*, **7**, 359–362.

MODJTAHEDI, H., STYLES, J., BOX, G., ECCLES, S., GUSTERSON, B. & DEAN, C. (1992). Antitumour activity of rat Mabs to the human receptor for EGF. In *Mutant Oncogenes: Targets for Therapy?* Epenetos, A.A. & Lemoine, N.R. (eds). Chapman & Hall, 1992 (in press).

MODJTAHEDI, H., STYLES, J.M. & DEAN, C.J. (1993). The human EGF receptor as a target for cancer therapy: six new rat MAbs against the receptor on the breast carcinoma MDA-MB 468. *Br. J. Cancer*, **67**, 247–253.

PELLEGRINI, R., CENTIS, F., MARTIGNONE, S., MASTROIONNI, A., TAGLIBUE, E., TOSI, E., MENARD, S. & COLNAGHI, M.L. (1991). Characterization of a monoclonal antibody directed against the epidermal growth factor receptor binding site. *Cancer Immuno. Immunother.*, **34**, 37–42.

RODECK, U., HERLYN, M., HERLYN, D., MOLTHOFF, C., ATKINSON, B., VARELLO, M., STEPLEWSKI, Z. & KOPROWSKI, H. (1987). Tumour growth modulation by a monoclonal antibody to the epidermal growth factor receptor: immunology mediated and effector cell-independent effects. *Cancer Res.*, **47**, 3692–3696.

SANTON, J.B., CRONIN, M.T., MACLEOD, C.L., MENDELSOHN, J., MASUI, H. & GILL, G.N. (1986). Effect of epidermal growth factor receptor concentration on tumorigenicity of A431 cells in nude mice. *Cancer Res.*, **46**, 4701–4705.

SATO, J.D., KAWAMOTO, T., LE, A.D., MENDELSOHN, J., POLIKOFF, J. & SATO, J.H. (1983). Biological effects in vitro of MAbs to human EGF receptors. *Mol. Biol. Med.*, **1**, 511–514.

SCHREIBER, A.B., LASX, I., YARDEN, Y., ESHHAR, Z. & SCHLESSINGER, J. (1981). Monoclonal antibodies against receptor for epidermal growth factor induce early and delayed effects of epidermal growth factor. *Proc. Natl Acad. Sci. USA*, **78**, 7535–7539.

SOO, K.C., WARD, M., ROBERTS, K.R., KEELING, F., CARTER, R.L., MCCREADY, V.R., OTT, R.J., POWELL, E., OZANNE, B., WESTWOOD, J.H. & GUSTERSON, B.A. (1987). Radioimmunoscintigraphy of squamous carcinomas of the head and neck. *Head & Neck Surg.*, **9**, 349–352.

SPORN, M.B. & ROBERTS, A.B. (1985). Autocrine growth factors and cancer. *Nature*, **313**, 745–747.

SPORN, M.B. & TODARO, G.J. (1980). Secretion and malignant transformation of cells. *N. Engl. J. Med.*, **303**, 878–880.

STECK, P.A., LEE, P. & HUNS, W.K. (1988). Expression of an altered epidermal growth factor receptor by human glioblastoma cells. *Cancer Res.*, **48**, 5433–5439.

VELU, T.J. (1990). Structure function and transforming potential of the epidermal growth factor receptor. *Mol. Cell. Endocrinol.*, **70**, 205–216.

WATERFIELD, M.D., MAYES, E.L.V., STROOBANT, P., BENNET, P.L.P., YOUNG, S., GOODFELLOW, P.N., BANTING, G.S. & OZANNE, B.A. (1982). Monoclonal antibody to the human epidermal growth factor receptor. *J. Cell. Biochem.*, **20**, 149–161.

WEBER, W., GILL, G.N. & SPIES, J. (1984). Production of an epidermal growth factor receptor – related protein. *Science*, **224**, 294–297.

YOSHIDA, K., KYO, E., TSUJINO, T., SANTO, T., NIMOTO, M. & TAHARA, E. (1990). Expression of epidermal growth factor transforming growth factor alpha, and their receptor genes in human gasteric carcinomas; implication for autocrine growth. *Jpn. J. Cancer Res.*, **81**, 43–51.